# Migration of endothelial cells on the surface of anodized Ni-Ti stent strut

**DOI:** 10.3389/fmedt.2023.1149594

**Published:** 2023-04-05

**Authors:** Zi Wang, Naofumi Ohtsu, Kasumi Tate, Yukiko Kojima, Hanif Saifurrahman, Makoto Ohta

**Affiliations:** ^1^ Institute of Fluid Science, Tohoku University, Sendai, Japan; ^2^ Graduate School of Biomedical Engineering, Tohoku University, Sendai, Japan; ^3^ Faculty of Engineering, Kitami Institute of Technology, Kitami, Japan; ^4^ Graduate School of Engineering, Tohoku University, Sendai, Japan

**Keywords:** endothelial cell, flow chamber, wall shear stress, endothelialization, anodization

## Abstract

**Background:**

Stent is widely regarded as the main treatment for curing cardiovascular diseases such as stenosis. Previous research has revealed that the damage of endothelial cells (EC), i.e., the components of endothelium, during stent implantation, could lead to severe complications, such as restenosis. To prevent restenosis, enhancements have been made to surface biocompatibility to accelerate the stent endothelialization process. Anodization on the Ni-Ti is a simple and efficient surface modification method to improve the biocompatibility of the Ni-Ti stent surfaces by enhancing the surface hydrophilicity, leading to an increase in the EC activities. The EC activity is known to be affected by the blood flow. Flow change by stent structure may result in EC dysfunctions, thereby leading to restenosis. It is thus essential to investigate the EC activities resulting from the anodization on the Ni-Ti surface under flow conditions.

**Objective:**

To study the influence of the endothelialization process on the Ni-Ti stent surface through anodization. The EC attachment and morphology on the anodized stent strut were observed under both with and without the flow conditions.

**Method:**

A parallel plate flow chamber was designed to generate a constant wall shear stress (WSS) to study the flow effect on the EC behavior. The hydrophilicity of the Ni-Ti stent strut surface was enhanced by a TiO_2_ layer fabricated *via* anodization. The EC distribution on the surface of the anodized nitinol stent strut was observed after 24 h of static (without flow) and flow exposure (with flow) experiment.

**Results:**

Under the static condition, the EC density on the surface of the anodized Ni-Ti stent strut was higher compared with the control. Under the flow condition, the enhancement of the EC density on the surface of the stent strut with anodization was reduced. The EC demonstrates a long and thin spindle-shaped morphology under the flow condition.

**Conclusion:**

Unlike the static condition, the EC is demonstrating a long and thin morphology in response to the flow under the flow condition. By improving the surface hydrophilicity, the anodization could enhance the EC migration onto the strut surface, and subsequently, accelerate the Ni-Ti stent endothelialization process. The improvement of the surface hydrophilicity is lower under the flow conditions when compared with the static conditions.

## 1. Introduction

Cardiovascular diseases (CVDs), such as stenosis and aneurysms, have emerged as one of the most life-threatening diseases worldwide. Stents have been used as the primary treatment for CVDs (1). The metal mesh structures are usually deployed at the target positions on the blood vessels to provide mechanical support to prevent the recoil of the blood vessel. However, the procedure of stent expansion occasionally creates endothelium lesion. The endothelium lesion could first trigger platelet aggregation to form a thrombus. Next, due to the deficient regulation of endothelial cells (ECs), the overproliferated smooth muscle cells (SMCs) can lead to the generation of neointima. Several *in-vivo* results have confirmed that EC dysfunction could alter its gene expression, resulting in abnormal conditions of the platelets and SMC. The thrombus and neointima could promote re-blockage in the vessel lumen (2–4). The negative effect of vascular re-blocking, such as restenosis and thrombosis, has become a severe complication after stent implantation (5–7). Therefore, the complications following the stenting treatment could be considered as an EC denudation around the stent struts. Increasing the adhesion and growth of EC on the surface of the stent strut, known as endothelialization, could reduce the vessel re-blockage.

EC forms the vascular innermost layer and is constantly exposed to the blood flow. To maintain vascular homeostasis, EC responds to the force generated by the blood flow, especially the wall shear stress (WSS) (8–10). Both *in-vivo* and *in-vitro* studies have revealed that the change in WSS could lead to EC dysfunctions, such as those related to the vascular cell adhesion molecules secreted by ECs (11, 12). WSS could also lead to the EC morphological response, such as orientation and elongation (13, 14). By using a parallel plate flow chamber with the presence of one stent strut within, Anzai et al. have observed that ECs migrate to the high WSS region (15). Moreover, owing to the flow being disturbed by the stent strut, the EC density differences on the side surfaces of the stent strut were also investigated. One more strut was added within the flow chamber by Wang et al. The ECs distribution is also in compliance with the WSS distribution (16). On the side surfaces of the stent strut, the EC density on the downstream side is higher than that on the upstream side, which demonstrates consistency with the previous study. These findings indicate that the structure of the stent strut could affect the flow near the EC layer, disturbing the flow changes in the WSS, and therefore, influence the EC activities around the stent strut.

To accelerate the process of endothelialization, several recent studies have attempted to modify the stent surface, including individually altering the chemistry and morphology of the surface, and both (17–21). The drug-eluting stents (DESs) are the stents coating drugs on the surface to modify the surface chemistry. DESs is revolutionized development followed by the bare metal stents. The drugs can prevent inflammation and proliferation of SMCs due to the vascular wall lesion caused by the stent implantation. However, side effects of DES, such as late thrombosis, delayed EC growth, and chronic inflammation, continue to persist as the risks of long-term restenosis (22). The modification of the surface morphology of the stent could improve the ECs activities, such as adhesion, migration, and elongation, and subsequently, could affect the endothelialization process for successful stent implantation. Several researchers have studied the effects of the Ni-Ti surface morphology, including the nanosized groove (19), pillar (20), tube (23), and pore (24). By using *in-vitro* models, researchers have discovered that the nanostructure could enhance the ECs attachment, spreading, and proliferation (19, 25). These investigations were performed without flow conditions for the most part. The effect of the flow on EC activity was not considered.

The anodization method aims to enhance the surface biocompatibility by altering the surface morphology of the titanium-based implants. With the advantage of a simple process, anodization could form a titanium dioxide (TiO_2_) layer with various morphologies and thicknesses in one step. The surface topography of TiO_2_ could be controlled by changing the combination of the applied voltage and electrolyte. The TiO_2_ layer could improve the surface hydrophilicity, and the nanosized pores on the surface could increase the cell's activation. Thus, the TiO_2_ layer acquired through anodization could enhance the biocompatibility of the surface. Yamasaki et al. found that the adhesion and proliferation of the MC3T3-E1 cells could be enhanced by the anodized surface (24). This result suggests that the cell activities could be enhanced by the TiO_2_ layer. The improvement of hydrophilicity through the anodization could increase the biocompatibility of the Ni-Ti sample surface. This enhancement could improve the EC activities, such as attachment and migration. Subsequently, it could accelerate the Ni-Ti stent endothelialization process.

It is well known that the ECs activity is affected by the flow, besides the static cell experiment, and it is necessary to evaluate the enhancement of the stent endothelialization process through the anodization treatment under the flow conditions. In this study, an *in-vitro* flow exposure experiment was performed by using a parallel plate flow chamber with the presence of the stent strut. The strut with anodization was chosen as an experimental group, and no-treatment and polished strut was the control. The objective of this study is to observe the EC migration and morphology on the surface of the Ni-Ti stent strut with anodization surface modification. This is the first study on the influence of the TiO_2_ nanostructure on an Ni-Ti stent strut formed by anodization on the EC activity under flow conditions.

## 2. Materials and methods

### 2.1. Preparation of the stent strut sample

#### 2.1.1. Samples

The Ni-Ti stent strut (0.4 mm * 0.4 mm * 18 mm) (Furukawa Electric Co., Ltd, Japan) without any treatment was prepared as the no-treatment control group. The Ni-Ti stent strut was firstly polished with a #4,000 emery paper and then ultrasonicated with ethanol and distilled water for 5 min each. The polished strut was connected to the anode, and the Ti plate was connected to the cathode as a counter electrode. The anodization was conducted using an electrolyte of 100 mM HNO_3_ aqueous solution. The temperature of the electrolyte was maintained at 20°C using a water bath. The galvanostatic direct current was applied during the anodization process at a constant current of 50 mA·cm^−2^ for 120 min. After completing the voltage applicant, the strut was ultrasonicated with distilled water for 5 min and dried in an ambient atmosphere. Hereafter, we refer to such samples as anodized samples.

In this study, no-treatment and polished samples were prepared as the two control groups. Ni-Ti stent strut with anodization treatment was referred as the experimental group. Apart from the abovementioned sample, we fabricated two types of Ni-Ti stent struts without anodization as the controls. One is the Ni-Ti stent without any surface treatment, including polishing, and the other is the surface-polished stent. We have named these samples as no-treatment and polished samples, respectively.

#### 2.1.2. Surface characterization

X-ray photoelectron spectroscopy (XPS; PHI-5000 VersaProbe, Ulvac-Phi, Japan) with a monochromatic Al Kα x-ray source (h*ν* = 1,486.6 eV) was used to analyze the chemical composition of the anodized stent surface. The photoelectron take-off angle was set to 45°. The survey spectrum with an energy resolution of 1 eV/step and narrow spectra with 0.2 eV/step were measured, sequentially. The formater spectrum is used for confirming the chemical composition, and the latter spectra was used for investigating the chemical state. The binding energies were calibrated by setting a hydrocarbon adsorbate to 284.8 eV.

Field-emission scanning electron microscopy (FESEM; JSM-6701F, JEOL Ltd., Japan) with a secondary electron mode was used to observe the surface morphology of the anodized stent surface. Scanning probe microscopy (SPM; SPM-9700, Shimadzu, Japan) was used to measure the surface roughness quantitatively. The topographic measurements were conducted using an atomic force microscopy (AFM) mode.

### 2.2. Cellular experiment

In this study, human carotid artery endothelial cells (Cell Applications, Inc) with passage numbers between 5 and 9 were used. The ECs were two-dimensional cultured in 35-mm culture dishes pre-coated with the gelatin solution (Wako Pure Chemical Industries, Ltd.) until reaching confluency. The proliferation medium for HCtAEC is Medium 199 (Gibco) containing 20% fetal bovine serum (Biosera), 1% penicillin/streptomycin (AUSTRAL Biologicals), and 0.001% human basic fibroblast growth factor (AUSTRAL Biologicals). The proliferation medium was used both in the cell culture and experiments.

#### 2.2.1. Without flow experiment

The experimental group was the stent strut with anodization surface treatment. The polished strut and no-treatment strut were the control group. The strut was implanted on the EC confluent monolayer in the culture dish. The EC images were collected after 24 h of static culture in the incubator.

#### 2.2.2. With flow experiment

The flow loop has been described in a previous study (15). The flow loop is with a pulse damper, flow chambers, a reservoir, and a roller pump. They were connected by silicone tubes. One Ni-Ti stent strut (0.406 mm * 0.406 mm * 18 mm) (Furukawa Electric Co., Ltd, Japan) was placed and covered with silicone gaskets (Fusogomu, Japan) at a thickness of 5 mm to fix the strut position within the parallel plate flow chamber during the experimental period of 24 h. The WSS in this study was 2 Pa generated by a roller pump with a volume flow rate of 2.16 × 10^−6^ m^3^/s. The gas (5% CO_2_ + 95% air) was connected to the flow loop to maintain the pH value of the working fluid. The temperature during the flow exposure is maintained constant at 37°C. The experimental setting of the two struts has been described in the previous study (16).

#### 2.2.3. Observation of EC fluorescent image

After 24 h of flow exposure, the ECs were fixed in 4% paraformaldehyde for 15 min at room temperature and then rinsed in phosphate-buffered saline. To permeabilize the ECs membrane, the cells were treated with 1 ml aliquots of 0.2% Triton X-100 (Roche Applied Science) for 5 min. The F-actin filaments of EC were stained with Alexa Fluor® 546 Phalloidin (Gibco), and the nuclei of the EC were stained with 4′, 6-diamidino-2-phenylindole (Gibco). The fluorescent images of EC were obtained through a microscope system (IX-83, Olympus, Japan).

ImageJ (NIH) was used to conduct image processing. The data for the ECs density and cell area are presented in the mean ± standard deviation (STD) format, where the mean represents the mean value of n measurements. The *T*-test was performed to compare the difference among groups. If the *p* value reported from the *t*-test is less than 0.05, then the result is said to be statistically significant.

## 3. Results

### 3.1. Surface characteristics of the anodized stent strut

Surface analysis of the anodized Ni-Ti surface was disclosed our previous study such as (25) in detail. Thus, we here provide only the important data relating with cell experiments.

**Figure 1:**
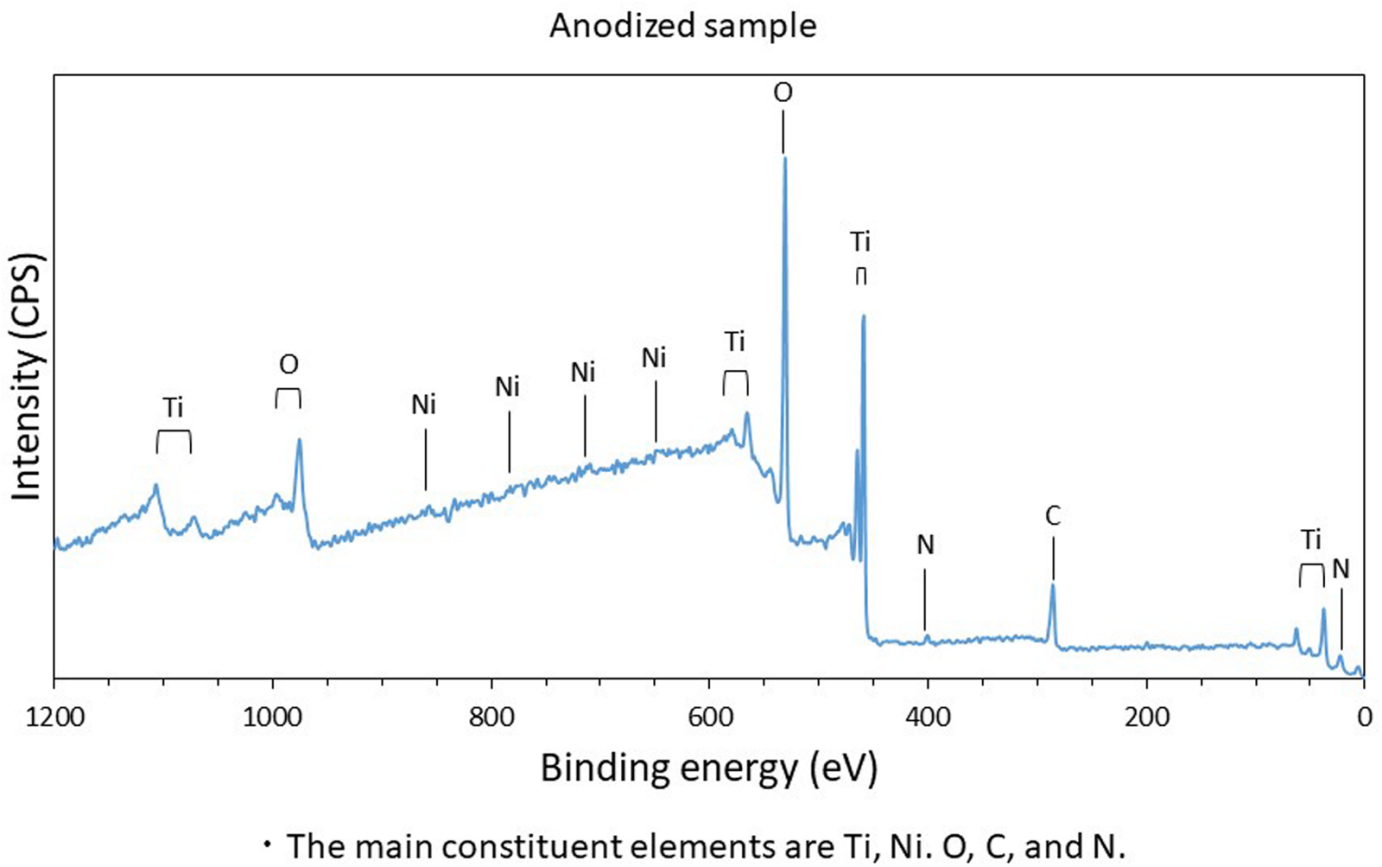
Constituent elements of anodized sample surface; the main constituent elements are Ti, Ni, O, C, and N.

**Figure 2:**
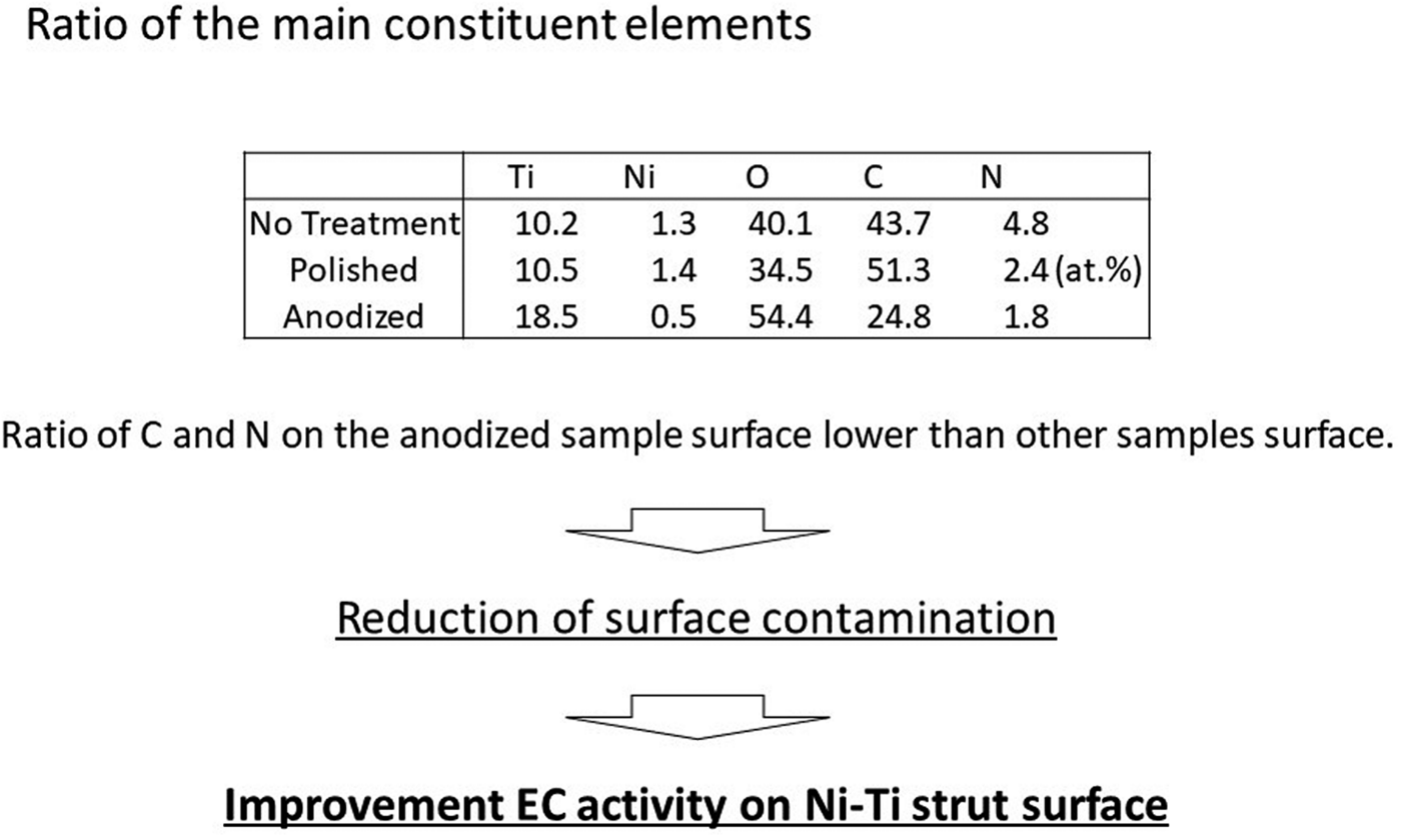
Atomic concentrations; the ratio of C and N on the anodized sample surface is lower than other samples surface.

As depicted in Figure 1, the XPS spectra obtained for the anodized stent revealed that the main constituent elements of the anodized stent are Ti, Ni, O, and C. The similar results were also observed in the spectra collected from no-treatment and polished samples. Hydrocarbon contaminants were the origin of carbon which were absorbed during exposure in an ambient atmosphere. The amount of such carbon adsorbate is known to relate with surface hydrophilicity. Also, the atomic concentration of Ni is below 5 at % as depicted in Figure 2, which is significantly lower than the nominal composition of the Ni-Ti stent. Further, the atomic ratio of O to Ti is ∼2, which is similar to that of TiO_2_. Further, the XPS narrow spectra for the Ti 2p and O 1s regions revealed that the chemical state of Ti and O corresponds to TiO_2_. Based on these results, the surface layer on the Ni-Ti strut formed by anodization is revealed to be TiO_2_, including a low concentration of Ni and hydrocarbon adsorbate.

Basically, the surface chemical state of the no-treatment and polished samples was similar to that of the anodized sample, whereas the amount of carbon adsorbate on the no-treatment and the polished surfaces was higher than that on the anodized surface. Further, the Ni concentration of the surface was also decreased by anodization. These results indicate that surface hydrophilicity of the Ni-Ti stent was improved through anodization, and additionally, the cytotoxic effect of the Ni on the stent was expected to be suppressed.

**Figure 3:**
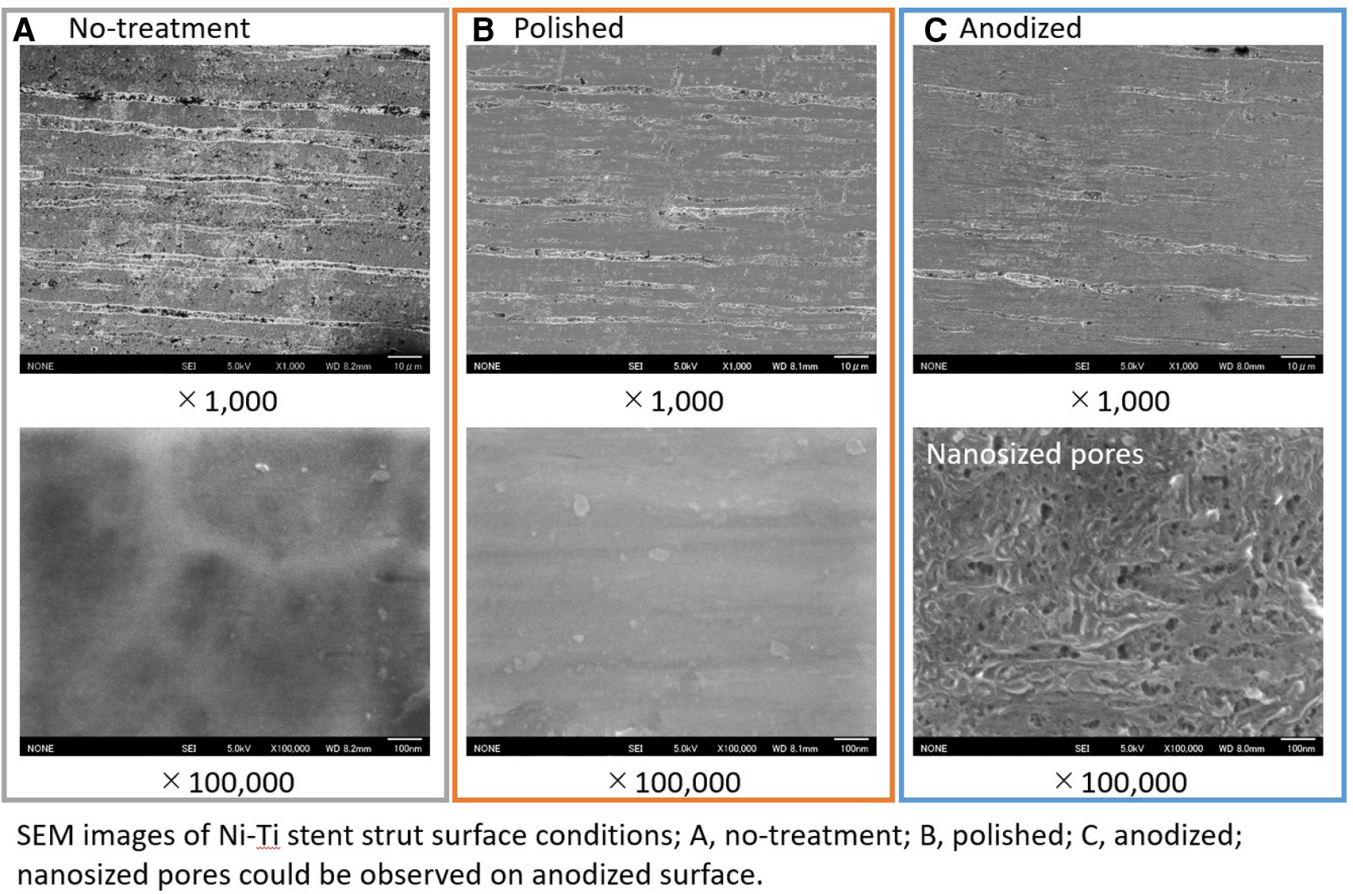
SEM images of stent strut surfaces; (**A**) no-treatment; (**B**) polished; (**C**) anodized.

The surface images obtained using FE-SEM are depicted in Figure 3. On comparison with the no-treatment Ni-Ti strut sample, the surface roughness was clearly reduced at the polished and anodized struts. The nanometer-sized pores were apparently observed on the anodized Ni-Ti strut sample. On the contrary, no such pores were observed on the polished and no-treatment samples. The principal of the formation of such nanosized pores are discussed in our previous study (25).

### 3.2. EC distribution

**Figure 4:**
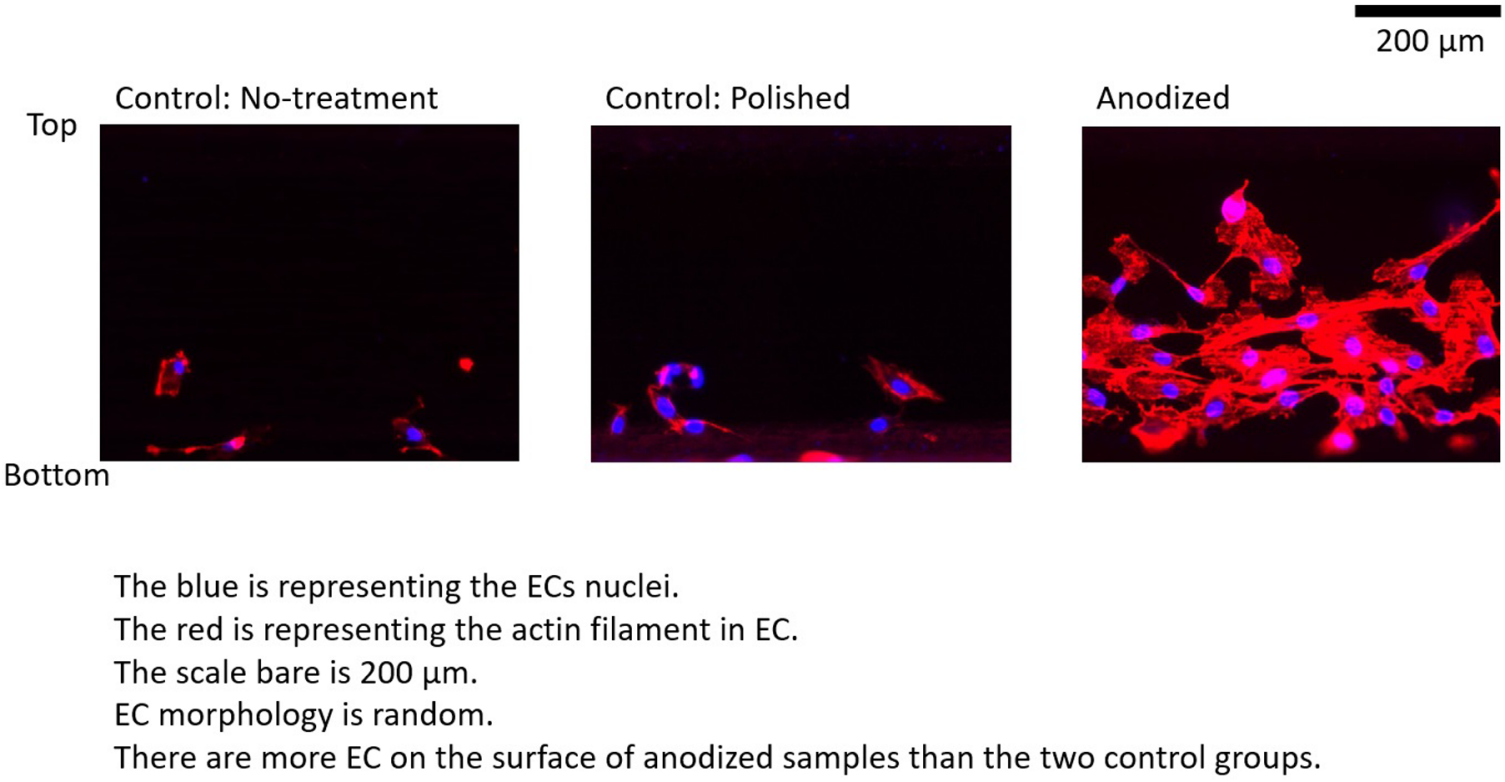
ECs on stent strut surfaces, without flow 24-h; blue color representing ECs nuclei; red color representing ECs actin filament; scale bar is 200 μm.

**Figure 5:**
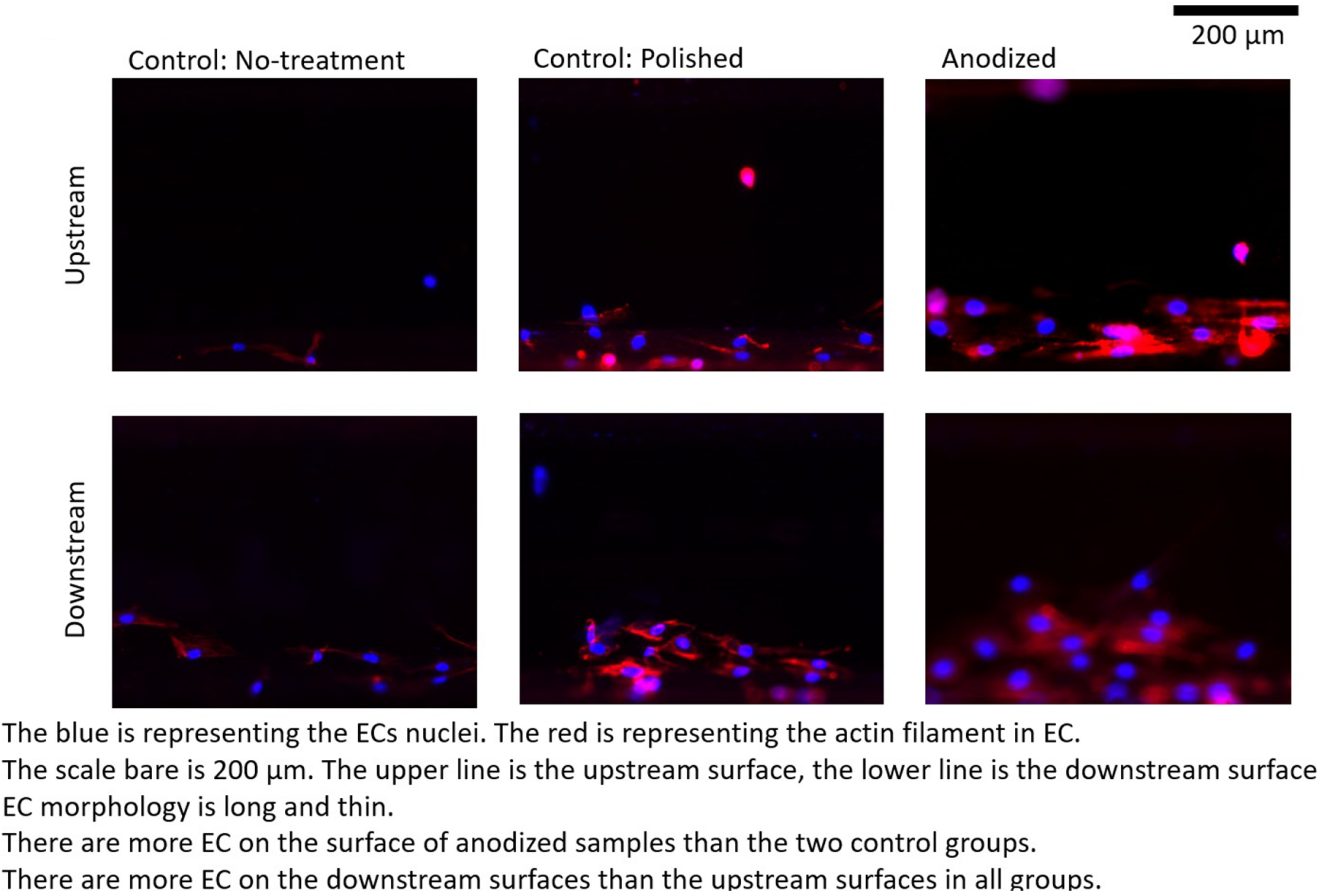
ECs on stent strut surfaces, with flow 24-h, one strut; blue color representing ECs nuclei; red color representing ECs actin filament; scale bar is 200 μm.

**Figure 6:**
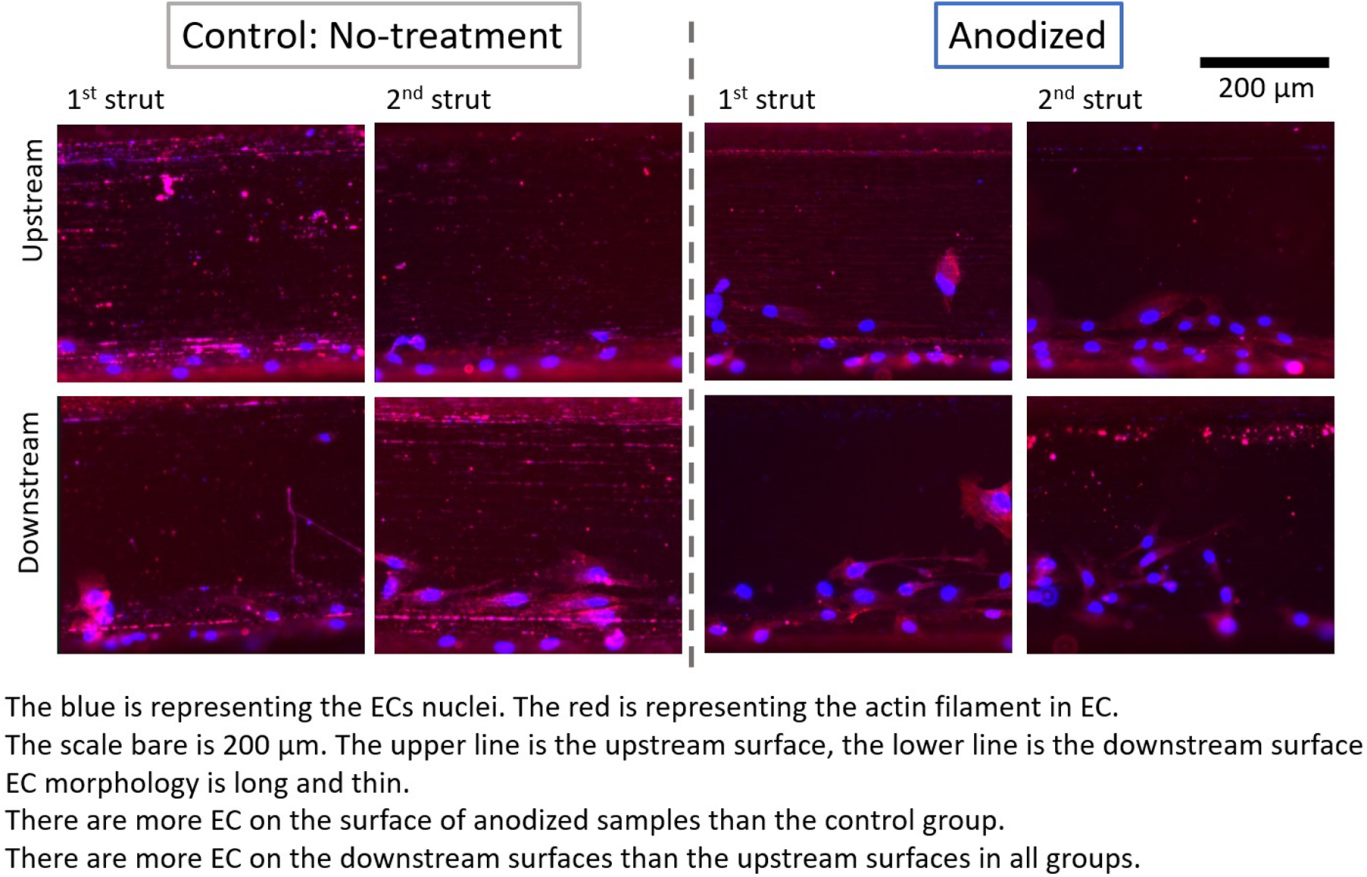
ECs on stent strut surfaces, with flow 24-h, two struts; blue color representing ECs nuclei; red color representing ECs actin filament; scale bar is 200 μm.

Figures 4–6 depict the ECs distribution on the surfaces of the stent strut. Under both with and without flow conditions, the ECs were highly attached near the bottom side than the top side. This phenomenon is caused by the EC migration from the bottom to the top of the Ni-Ti strut side surfaces. The higher position of EC migration onto the side surfaces of the stent strut could be detected on the anodized surfaces compared to both the control groups. Moreover, the migration distance on the without flow condition is higher than that with the flow condition. Additionally, the number of EC on the anodized strut surface is higher than both the control groups. In the static group, the EC depicts a random morphology. In the with flow group, compared with the static group, the EC demonstrates a long and thin spindle-shaped morphology. This is because of the EC response to WSS stimuli. There are more cells on the downstream surfaces than the upstream surfaces in both the experimental and control groups.

By analyzing the nuclei (blue color in the ECs distribution figures) quantitatively, the EC density on the side surfaces of the stent strut could be obtained. EC density analysis could be used to evaluate the number of EC migrate from the bottom surface on the culture dish to the side surface of the stent strut, then to evaluate the process of endothelialization. In addition, this study analyzed the area of the actin filament (red color in the ECs distribution figures). The area of actin filament was used to represent the EC area. EC area could be considered as a parameter to measure the EC activity.

### 3.3. EC density

**Figure 7:**
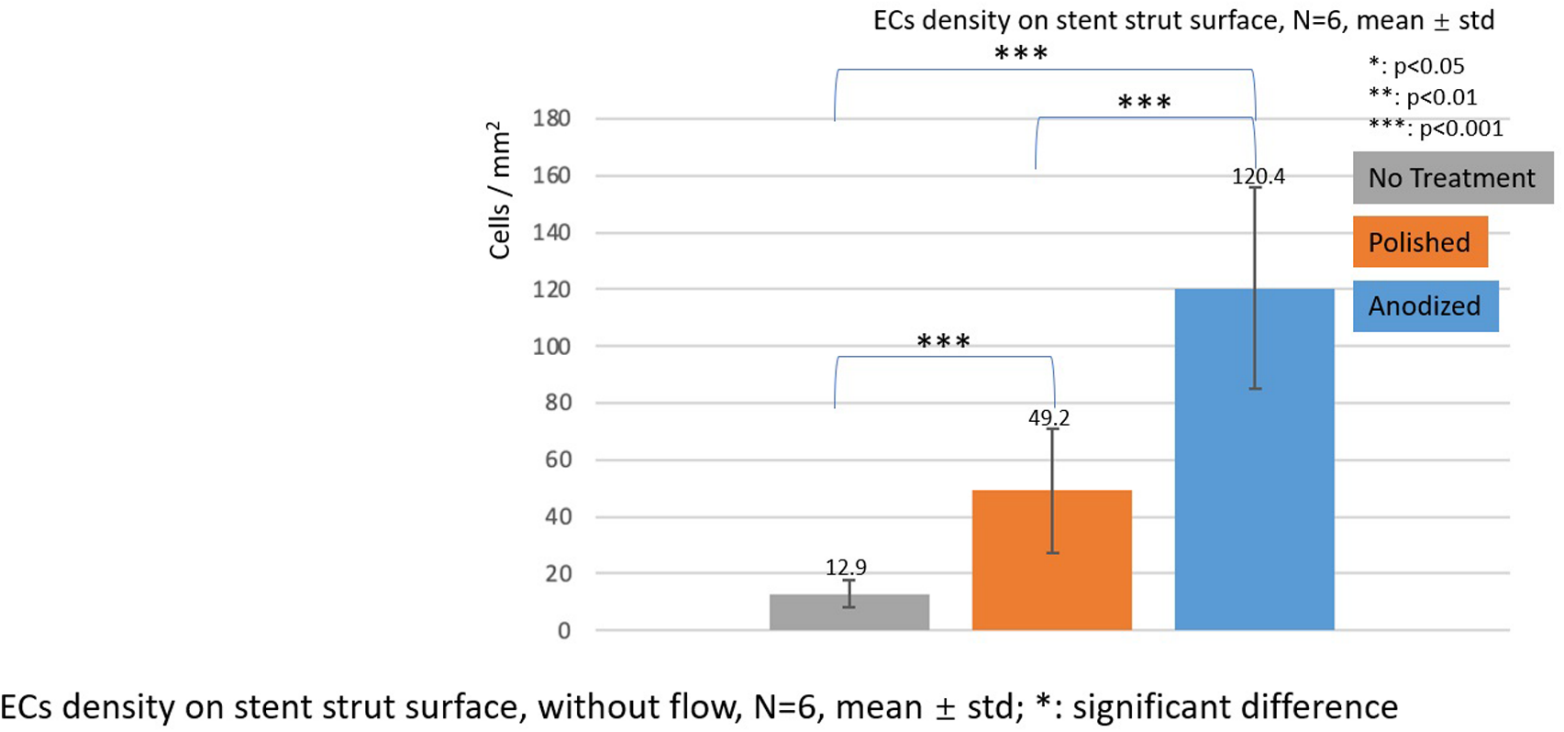
ECs density on stent strut surfaces, without flow 24-h, *N* = 6, mean ± std; *T*-test was performed to compare the significant difference (*p < 0.05; **p < 0.01; ***p < 0.001).

**Figure 8:**
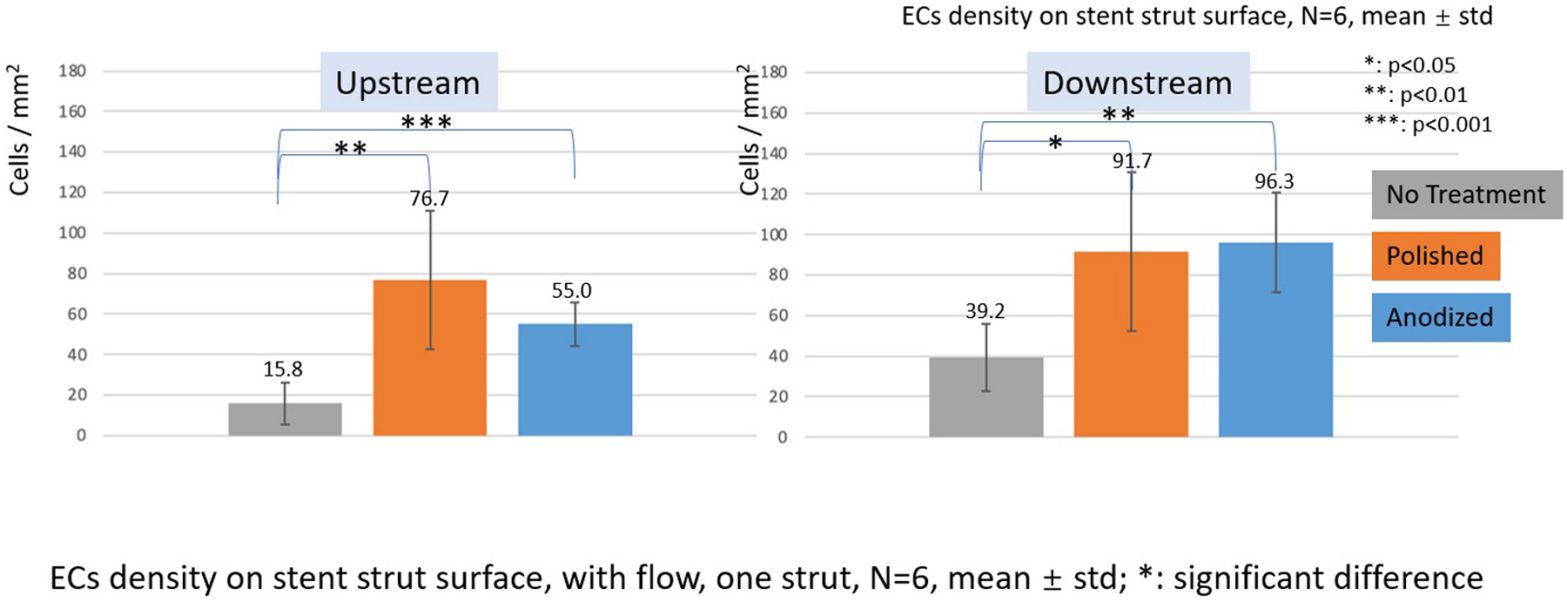
ECs density on stent strut surfaces, with flow 24-h, one strut, *N* = 6, mean ± std; *T*-test was performed to compare the significant difference (*p < 0.05; **p < 0.01; ***p < 0.001).

**Figure 9:**
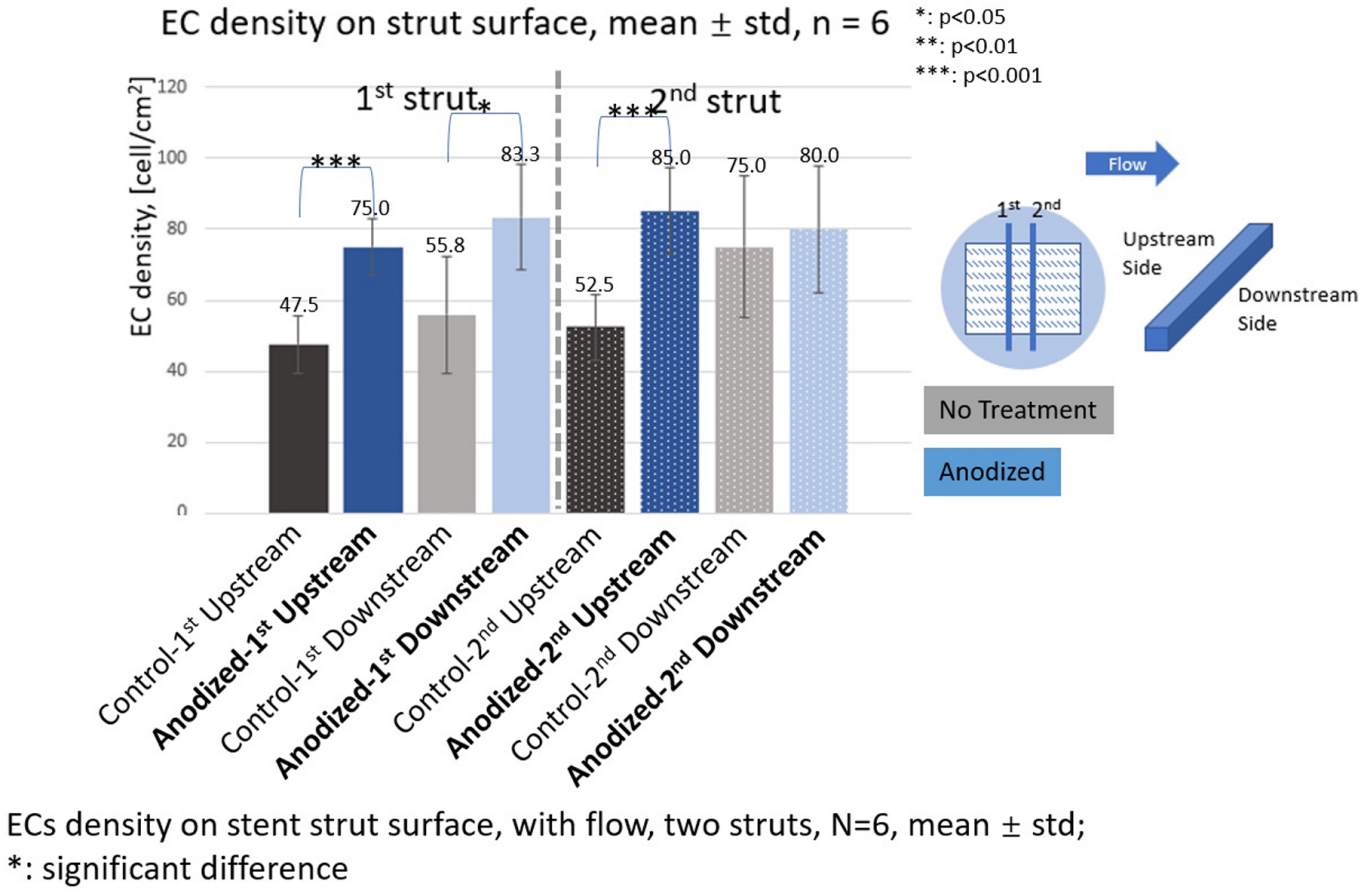
ECs density on stent strut surfaces, with flow 24-h, two struts, *N* = 6, mean ± std; *T*-test was performed to compare the significant difference (*p < 0.05; **p < 0.01; ***p < 0.001).

Figures 7–9 depict the ECs density on the strut surface. In the without flow group, the EC highest density is on the anodized strut surface, while the lowest density is on the no-treatment surface in the control group. There exist significant differences among the three groups.

In the with flow cases, the EC lowest density is on the no-treatment surfaces on both the upstream and downstream surfaces, and a significant difference was detected between the other two groups.

With the presence of one stent strut, on the upstream surfaces, the highest EC density is on the polished surface. On the downstream surfaces, the highest EC density is on the anodized surface. However, there is no significant difference between EC density on anodized surfaces and polished surfaces.

With the presence of two stent struts, the EC density on both the upstream and downstream side surfaces of the second strut is higher than that on the first strut, except on the downstream side surfaces of the second anodized stent strut.

### 3.4. EC area

**Figure 10:**
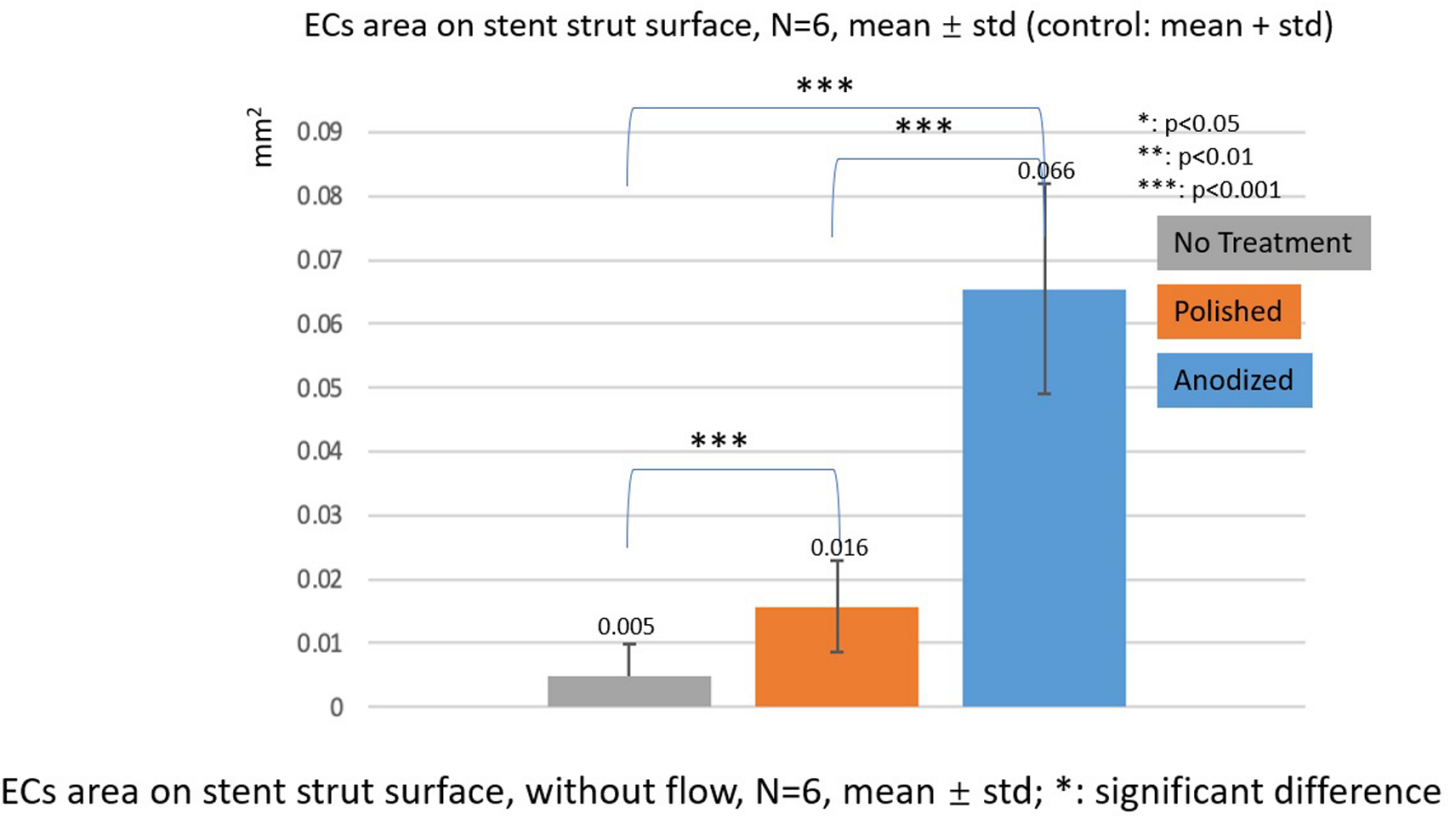
ECs area on stent strut surfaces, without flow 24-h, *N* = 6, mean ± std; *T*-test was performed to compare the significant difference (*p < 0.05; **p < 0.01; ***p < 0.001).

**Figure 11:**
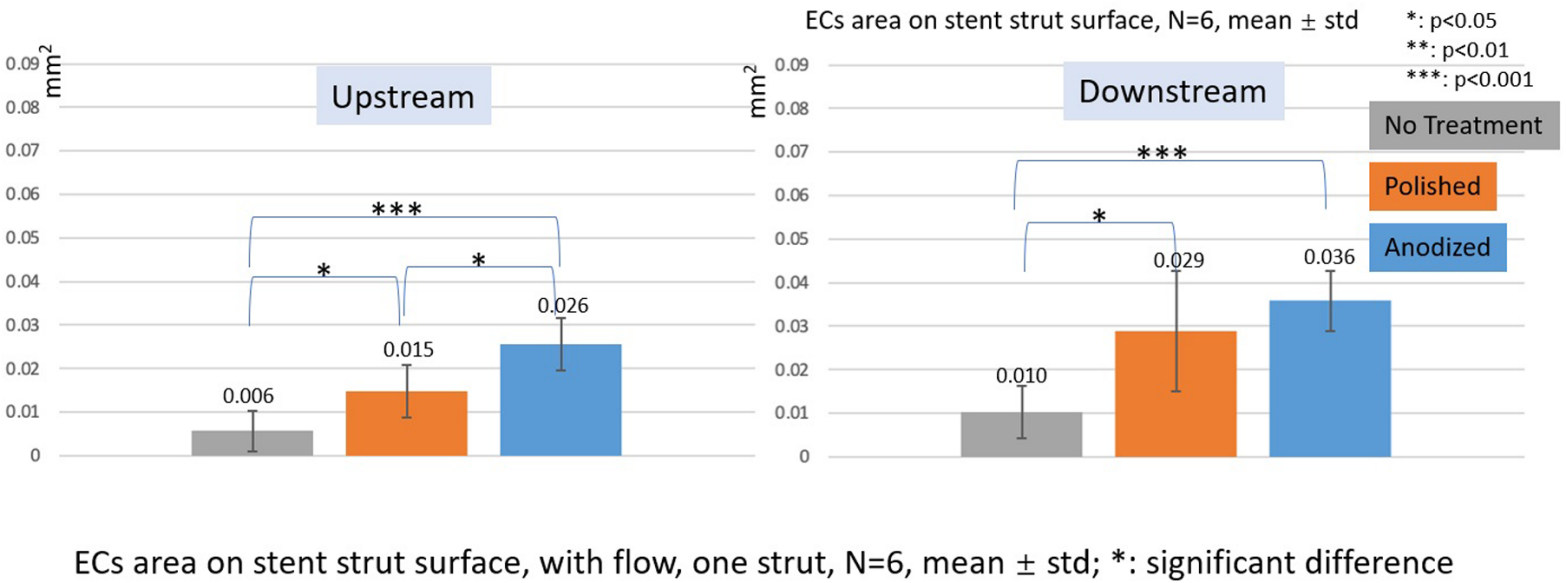
ECs area on stent strut surfaces, with flow 24-h, one strut, *N* = 6, mean ± std; *T*-test was performed to compare the significant difference (*p < 0.05; **p < 0.01; ***p < 0.001).

Figures 10, 11 depict the ECs area on the strut surface. Under the without flow conditions, the EC largest area is on the anodized surface, while the smallest area is on the no-treatment surface in the control group. There are significant differences among the three groups.

In the with flow cases, the tendency of EC area is similar as the without flow cases. The smallest area is on the no-treatment surfaces, and the largest area is on the anodized surfaces on both upstream and downstream surfaces of the stent strut. There are significant differences among the three groups, except for the anodized and polished groups on the downstream surface.

## 4. Discussion

This study describes the EC distribution on the surface treatment effect of the Ni-Ti stent strut under both with and without the WSS experimental conditions on the EC activities. The EC density and morphology were observed to measure the stent endothelialization process enhanced by the anodization treatment.

As shown in the figures of the results, a higher EC density on the surfaces of the stent strut was observed on the anodized experimental group than the other two control groups (polished and no-treatment) under both with and without flow conditions.

It is considered that surface roughness and hydrophilicity affect cell activities (26–29). The samples in this study were prepared with no-treatment, polishing, and anodization followed by the procedure to anodization. The polishing reduced the surface roughness. After this polishing procedure, the anodization treatment was performed, and the hydrophilicity of the stent strut surface was improved due to the formation of the TiO_2_ layer. Although the surface roughness was modified by the TiO_2_ layer leading nanopores, the surface roughness of the anodized sample on a micron-size was almost similar to the polished one as shown in Figure 3. Yamasaki et al., pointed out that the anodization modified the surface hydrophilicity (24). Additionally, several papers, including Ohtsu, pointed out that the reduction of surface roughness and hydrophilicity of TiO_2_ will increase the cell activities (30–32).

Owing to the size limitation of the stent strut, it is difficult to measure the thickness of the TiO_2_ layer and surface hydrophilicity of the anodized strut. The setting of the anodization is the same as the one reported in “N10P0” by Yamasaki et al., and therefore, the thickness of the TiO_2_ layer of the anodized strut could be 120 nm. The surface hydrophilicity represented by the water contact angle could be approximately 10 degrees (24).

Under the static condition, the highest EC density was observed on an anodized stent strut surface. Moreover, the polished strut revealed the next highest density, and the difference among the anodized, polished, and no-treatment strut were significant. These results are similar and consistent with those reported in a previous study by Yamasaki et al. (24). The reason for the difference in the EC density between the anodization experimental group and both the control groups may be because the reduction of the surface roughness increases the EC adhesion, and the improvement of the surface hydrophilicity through anodization may further enhance the EC migration onto the strut surface.

Under the flow conditions with the presence of one stent strut, a higher EC density and a significant difference could be found on the anodized strut side surfaces compared with the no-treatment control group. However, the EC density on the anodized strut, especially the upstream surface, was lower than that of the polished control group. Overall, if the sum of the cell density of both the upstream and downstream surfaces is considered as a whole, there is no significant difference of EC density between the anodization group and the polished control group. This reduction in the ECs density of the anodized group may occur due to the flow.

The WSS is generated by the flow and affects the cell activities (33–35). Moreover, Wang et al. have demonstrated that the stent struts disturbed the flow and altered the WSS distribution (15, 16). The results of the cell density difference among the three samples lead to the effect of WSS on cell activities. The WSS effect is dependent on the surface roughness and hydrophilicity. The hydrophilicity enhanced by the anodization might be suppressed by the flow. For this reason, the attraction of the anodized surfaces to the EC migration is reduced.

Under the flow condition with the presence of the strut, the EC density on the downstream surfaces is higher than that on the upstream surfaces in the anodization group. This tendency of the appearance of the higher EC density on the downstream side is consistent with the results of our previous study. With the presence of the two struts in the anodization group, the EC density on the downstream surfaces of the second strut is lower than that on the downstream surfaces of the first strut. If the mean value of the EC density on the downstream surfaces of both the struts could be regarded as a whole, the mean value of the EC density on the downstream surfaces in the two struts is lower than that in case of the downstream surface in one strut. Compared to the upstream side, the flow is more complex around the downstream side due to the strut structure disturbance. The enhancement of EC migration in the anodization group could be further suppressed by the complex WSS generated by the flow.

In this study, we also used the EC area as a parameter to evaluate the EC activity as depicted in Figures 10, 11, although other previous studies have used the EC density as an evaluation of the EC activity (36–38). The results of the EC area were different from those of the EC density, especially on the upstream side in the case of one strut. On the upstream side, the EC area on the anodized surfaces was significantly higher than that of the polished one, whereas the EC density on the anodized surfaces was lower than that of the polished surfaces.

### 4.1. Limitation

The current experimental setting of the flow condition is 2 Pa steady since it is the start-up phase to study the effect of the flow on the EC activities. The 2 Pa WSS is in the range of normal vascular WSS. Below or above the normal value of WSS could lead to the physiological condition. EC under physiological WSS condition could change its alignment, elongation, and gene expression (34, 39). The different settings of the WSS value, and flow conditions such as the pulsatile flow may affect the EC activities mentioned in the refs (40–42).

## 5. Conclusion

This research analyzed the ECs behavior on the surface of the Ni-Ti stent strut with and without the anodization surface treatment. The experiment was conducted under static and flow conditions. In the flow exposure experiment, one and two Ni-Ti struts were placed within the parallel plate flow chamber. Under the flow condition, the EC demonstrates a long and thin spindle-like morphology in response to the flow stimuli, whereas the EC demonstrates a random shape under the static condition. A higher EC density on the strut surface was found at the anodized strut under both with and without flow conditions. However, on anodized samples, the attraction effect on EC migration could be suppressed by the flow. Due to the enhancement of the biocompatibility of the Ni-Ti surface due to anodization, EC migration onto the stent strut surface could be promoted, and subsequently, may accelerate the endothelialization process.

## Data Availability

The original contributions presented in the study are included in the article/**Supplementary Material**, further inquiries can be directed to the corresponding authors.
