# Supplementary material for: Migration of endothelial cells on the surface of anodized Ni-Ti stent strut
Source: Front Med Technol. 2023 Apr 5;5:1149594. doi: 10.3389/fmedt.2023.1149594 (PMC10113440; doi:10.3389/fmedt.2023.1149594)
Supplement: Supplementary file 1 [file Datasheet1.pdf]

Surface hydrophilicity measurement

The surface hydrophilicity was represented by water contact angle. The contact angle against distilled water was measured using a contact angle meter (DM-CE1, Kyowa Interface Science, Japan).

Figure 1 shows the wettability measurement on the surface of the stent strut. Figure 2 shows the quantitative analysis of the water contact angle. The water contact angle is similar between no-treated and polished stent strut surface. The TiO_2 layer obtained by anodization significantly increase the hydrophilicity.

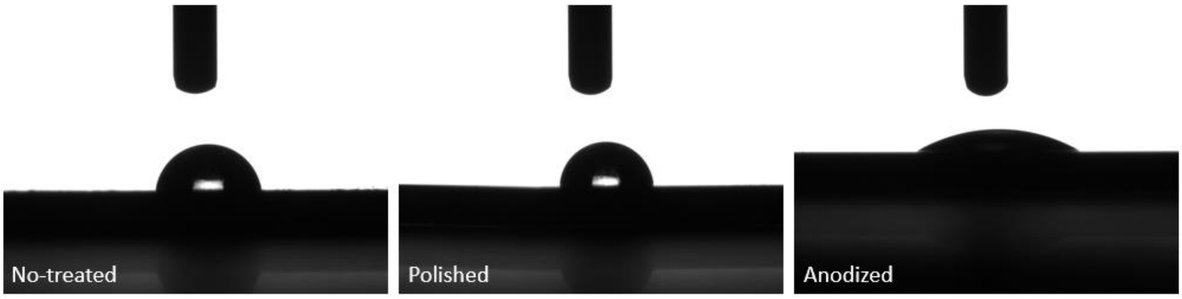

Figure 1, Wettability measurement.

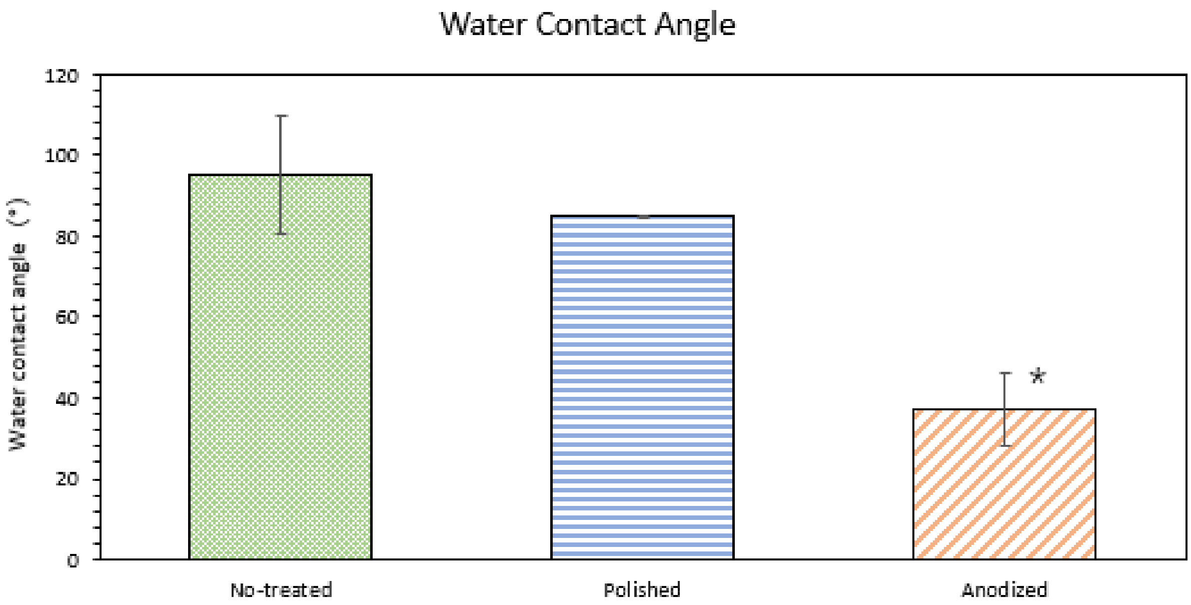

Figure 2, Water contact angle analysis, mean ± std, *, significant difference.
